# National interest may require distributing COVID-19 vaccines to other countries

**DOI:** 10.1038/s41598-021-97544-5

**Published:** 2021-09-14

**Authors:** Tiziano Rotesi, Paolo Pin, Maria Cucciniello, Amyn A. Malik, Elliott E. Paintsil, Scott E. Bokemper, Kathryn Willebrand, Gregory A. Huber, Alessia Melegaro, Saad B. Omer

**Affiliations:** 1 Department of Economics, University of Lausanne, Lausanne, Switzerland; 2 Department of Economics and Statistics, Università Di Siena, Siena, Italy; 3 Bocconi Institute for Data Science and Analytics (BIDSA), Bocconi University, Milan, Italy; 4 University of Edinburgh Business School, Edinburgh, Scotland, UK; 5 Dondena Centre for Research in Social Dynamics and Public Policy, Bocconi University, Milan, Italy; 6 Yale Institute for Global Health, New Haven, CT USA; 7 Yale School of Medicine, New Haven, CT USA; 8 Columbia University, New York, NY USA; 9 Institution for Social and Policy Studies, Yale University, New Haven, CT USA; 10 Center for the Study of American Politics, Yale University, New Haven, CT USA; 11 Yale School of Public Health, New Haven, CT USA; 12 Department of Political Science, Yale University, New Haven, CT USA; 13 Department of Social and Political Sciences, Bocconi University, Milan, Italy; 14 Yale School of Nursing, Orange, CT USA

**Keywords:** Epidemiology, Vaccines, SARS-CoV-2

## Abstract

As immunization campaigns are accelerating, understanding how to distribute the scarce doses of vaccines is of paramount importance and a quantitative analysis of the trade-offs involved in domestic-only versus cooperative distribution is still missing. In this study we use a network Susceptible-Infected-Removed (SIR) model to show circumstances under which it is in a country’s self-interest to ensure other countries can obtain COVID-19 vaccines rather than focusing only on vaccination of their own residents. In particular, we focus our analysis on the United States and estimate the internal burden of COVID-19 disease under different scenarios about vaccine cooperation. We show that in scenarios in which the US has reached the threshold for domestic herd immunity, the US may find it optimal to donate doses to other countries with lower vaccination coverage, as this would allow for a sharp reduction in the inflow of infected individuals from abroad.

## Introduction

The COVID-19 pandemic has caused enormous morbidity and mortality globally with over 184 million cases and almost 3.9 million deaths as of July 2021^1^. In the absence of a vaccine or substantively effective pharmacological interventions, governments mostly relied on stay-at-home orders and border lockdowns to slow the spread of the disease^2^. However, this has resulted in disruption of many economic activities with resulting financial losses and long-term consequences^3–7^.

As the first vaccines are now available, scientists have turned to the key question of how to distribute scarce supplies. While a great deal of this focus has been on prioritization *within* countries, there is a perhaps even more important question about how to allocate the vaccine *across* countries. In fact, the optimal strategy for securing vaccine supplies and balancing the global interest with individual countries’ self-interest is unclear. As the discussion on equitable vaccine distribution continues, some countries have already reserved significant doses of vaccines for themselves^8^.

The World Health Organization launched the COVAX pillar of the Access to COVID-19 Tools Accelerator to provide a global solution and equitable access to vaccines^9^. The COVAX Facility aims to support the development and manufacturing of various COVID-19 vaccine candidates and negotiate pricing. The COVAX Advance Market Commitment (AMC) is an important component of the COVAX Facility that will provide vaccines to 92 low and lower-middle income countries through a unique funding mechanism leveraging the required volume and buy-in from higher income countries participating in the program. However, not all countries are participating in the COVAX Facility. A few countries have struck individual deals with the manufacturers most likely to produce an effective vaccine, resulting in concerns about ‘vaccine nationalism’^10^. This country-by-country approach ignores the fact that ending a global pandemic requires all countries to have sufficient access to the vaccine for their populations.

Because closing international borders^11^ over the long-term is likely not an economically viable option, it may be in the self-interest of countries that have greater access to vaccines to cooperate and share doses with others. The home country-first approach also carries a risk that if a vaccine manufacturer is not successful, countries signing deals only with them may be locked out of a vaccine. However, whether, and under which conditions, it is in a resource rich country’s interest to support vaccine allocation to other countries is not currently known.

## The model

To study the effect of cooperative versus uncooperative vaccine distribution behavior, we use a network model where countries are nodes and links are individuals moving from one country to another. For each country, a separate compartmental SIR model was constructed, whereby individuals can become infected through interaction with SARS-Cov-2 positive cases under the assumption of internal homogeneous mixing. Network effects are such that the safety of one country also depends on the risk of having large shocks in other parts of the world, especially in those countries where herd immunity levels have not been achieved. Specifically, if there is an outbreak in one country, infected individuals may spread it to other countries through the modeled mobility patterns. In the Methods section below, we describe the mechanics of our modeling approach. To quantify the impact of these effects between countries, we calibrate our model to real data.

We model international travel using data on air travel and cross-border ground travel. Airline transportation data gathered by the International Civil Aviation Organization (ICAO) allows us to estimate the average daily number of passengers from each country of origin to the rest of the network. Ground mobility flows from the US to both Mexico and Canada, and vice versa, are used to estimate those travel patterns (Supplementary Fig. S1 represents the mobility network).

**Figure 1:**
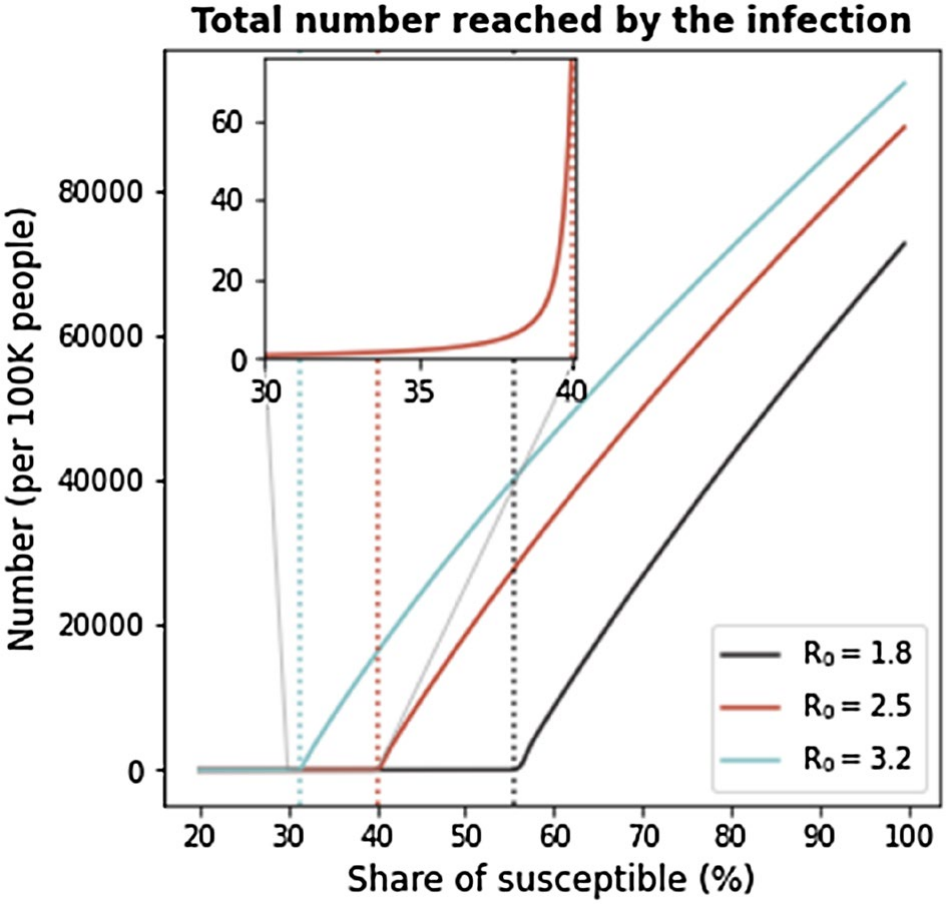
Number of infected individuals as a function of the share of susceptible in the population. When the proportion of susceptible individuals crosses the herd immunity threshold (1/*R*_0_), the number of infections increases drastically. Dotted lines represent herd immunity levels associated with three levels of *R*_0_.

As we elaborate below in the Methods sections, in a standard SIR framework, herd immunity is achieved when the share of protected individuals is above a certain threshold $P_{c} = 1 - 1/R_{0}$ ^12^. The higher the average number of secondary cases that can be generated by one single infectious case $\left( {R_{0} } \right),$ the larger the proportion of individuals in a population who must be vaccinated to reach the herd immunity level. This is particularly relevant when studying the number of individuals who become infected in the presence of a single primary case (Fig. 1). When one infectious person appears in a given population, the number of secondary cases changes as a function of how many in the population are susceptible. When a country is close to the threshold of herd immunity, there is a clear discontinuity in the number of cases that follow from a new infection, as the effect of a shock increases by several orders of magnitude once herd immunity is lost.

This implies that once a country is above herd immunity, it becomes crucial for their self-interest to help other countries reach that goal. The reason for this is that increasing vaccination rates at home will only mildly affect the expected number of new infections if a new wave arrives, but increasing vaccinations abroad for countries approaching the herd immunity threshold will substantially reduce the risks of waves from abroad arriving domestically. This, in turn, has a much greater impact, even on new domestic infections.

## Results

We start by defining a measure of risk, which captures the degree of exposure of each country to infections from the rest of the network (see Methods section) and we find that the US is particularly exposed (Supplementary Fig. S2) because of high levels of cross border travel. Focusing on the US, we performed simulations assuming that an infection starts somewhere else in the world. This scenario is representative of a situation in which a population with low immunity levels experiences an internal outbreak that may then travel to other countries through individuals’ mobility patterns. We then estimate how many people we expect to get infected in the US.

Two counterfactual alternative vaccines allocation strategies are evaluated. One is a domestic-only allocation strategy and in the other the US donates some doses of its vaccine stock to other countries. Specifically, we assume that the US has available a number D of extra doses of a vaccine with a modeled efficacy of 95%. These doses could either be kept for domestic distribution (uncooperative strategy) or else distributed in any other country (cooperative action) in the group of low-income countries belonging to COVAX Advanced Market Commitment (henceforth COVAX-LIC). To compare the output of the cooperative and the uncooperative action, we run simulations of our model and estimate the difference in the overall number of domestic cases in the US. The framework of our analysis can be applied to an initial period of vaccine allocation, but also to a later condition in which a country has allocated some doses domestically and is getting D additional doses.

We note that this is a hard test of the claim that cooperative vaccine distribution is desirable. While sharing a scarce vaccine stock with other countries will reduce deaths in those receiving countries, we ignore those welfare effects and instead concentrate narrowly on whether vaccine sharing is good for the donating country rather than the recipient because self-interest is likely to be a strong motivation for embracing the cooperative policy. Roughly, is it in the self-interest of the US to share its scarce vaccine asset to minimize domestic mortality?

**Figure 2:**
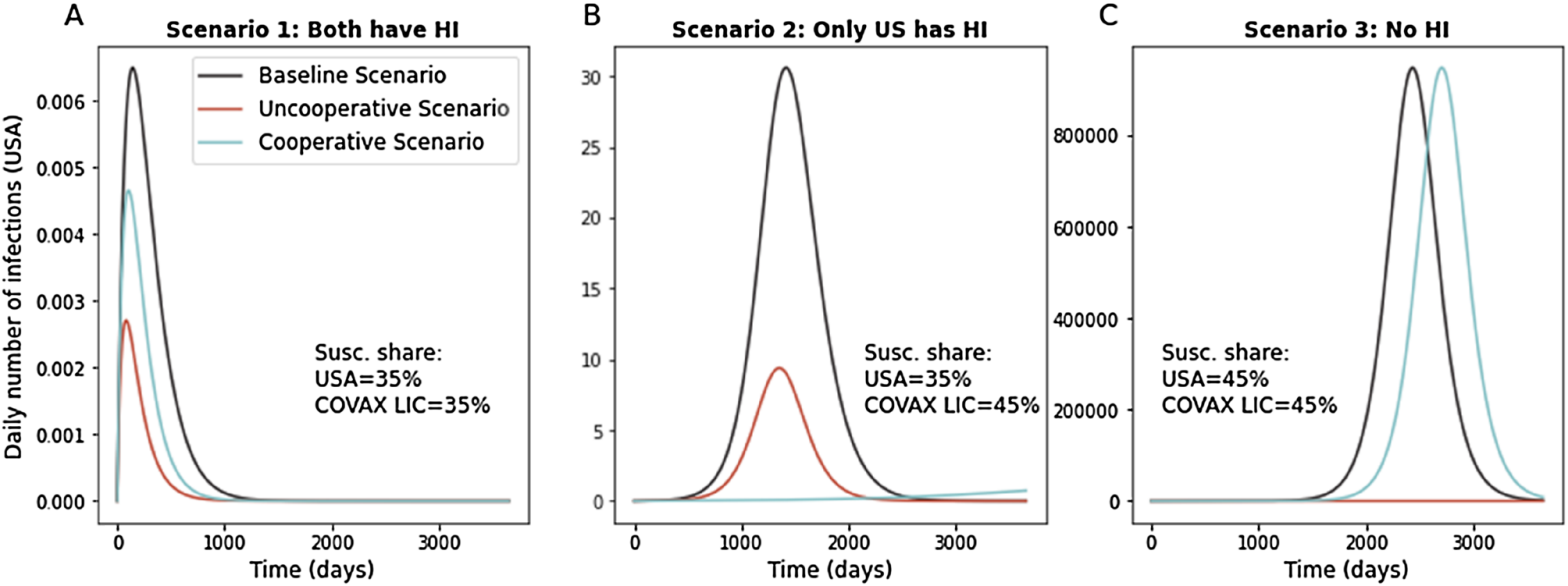
Daily number of infections in the US, comparison between Uncooperative and Cooperative scenarios. The figure compares between distributing 60mln extra doses in the USA (uncooperative scenario: red line) or in COVAX-LIC (cooperative scenario: light blue). In the baseline scenario (black line) the extra doses are not distributed. All panels consider *R*_0_ = 2.5 and the initial share of susceptible in the rest of the world equal to 35%. (**A**) The figure shows the daily number of infections in the US as estimated by the SIR model, when at time 0 the number of infected individuals equals 1000 in COVAX-LIC and is 0 everywhere else. The share of susceptible is 35% in both the US and in the COVAX-LIC. (**B**) As (**A**), but with initial shares of susceptible equal to 35% in the US and 45% in COVAX-LIC. **(C)** As (**A**), but with initial shares of susceptible equal to 45% in the US and 45% in COVAX-LIC.

Figure 2 provides three examples, each connected to a different qualitative behavior of the system. In these simulations we focus on the US and on COVAX-LIC (for the list of countries see supplementary materials). We fix ${R}_{0}$=2.5 and we keep the rest of the world with susceptibility equal to 35%, which means that these countries are above herd immunity levels and so they do not contribute significantly to the spread of the disease. We then consider an initial outbreak of 1000 new infections taking place in COVAX-LIC at time 0 and we follow the spread of the disease in the US under three scenarios. The baseline scenario considers the case of not having any extra doses of vaccine. The uncooperative scenario models the case in which 60 million extra doses are distributed in the US (we consider 2 doses of vaccine to be necessary for 95% efficacy). Finally, the cooperative scenario studies the case in which 60 million doses are given to COVAX-LIC (cooperative strategy). The first panel on the left considers the case in which both the US and COVAX-LIC countries have 35% of their population susceptible to COVID-19 before distributing the vaccine. As both countries have achieved herd immunity, we see the impact on the US is negligible. The extra doses marginally reduce contagion, but to a degree that can be approximated to 0 (note the very small scale of the vertical axis). This is because the disease does not spread exponentially either domestically or abroad. In the central panel of Fig. 2 we see the case in which the US starts with a share of susceptible equal to 35%, while COVAX-LIC countries have 45% of their population at risk (below herd immunity). In this case, in COVAX-LIC countries the disease would spread to 5% of the population without extra doses of vaccine, and then begin to spread to the United States. When an outbreak takes place without additional vaccinations, the number of cases in the US peaks sharply, while it is suppressed by about 2/3rd when the additional vaccine doses are allocated domestically. However, allocating the additional doses cooperatively to the COVAX-LIC countries avoids this spillover all together by preventing the foreign outbreaks that would later spread to the US. In this case we therefore show that cooperation actions would allow for a reduction in the spread of the disease *in the US*. Finally, the panel on the right depicts a case in which both the US and the COVAX-LIC countries have relatively high rates of vulnerability and are thus below herd immunity. In this case, while allocating the doses to other countries delays the arrival of cases in the US, domestic allocation is more efficacious for protecting the domestic population because it allows the US to achieve herd immunity.

**Figure 3:**
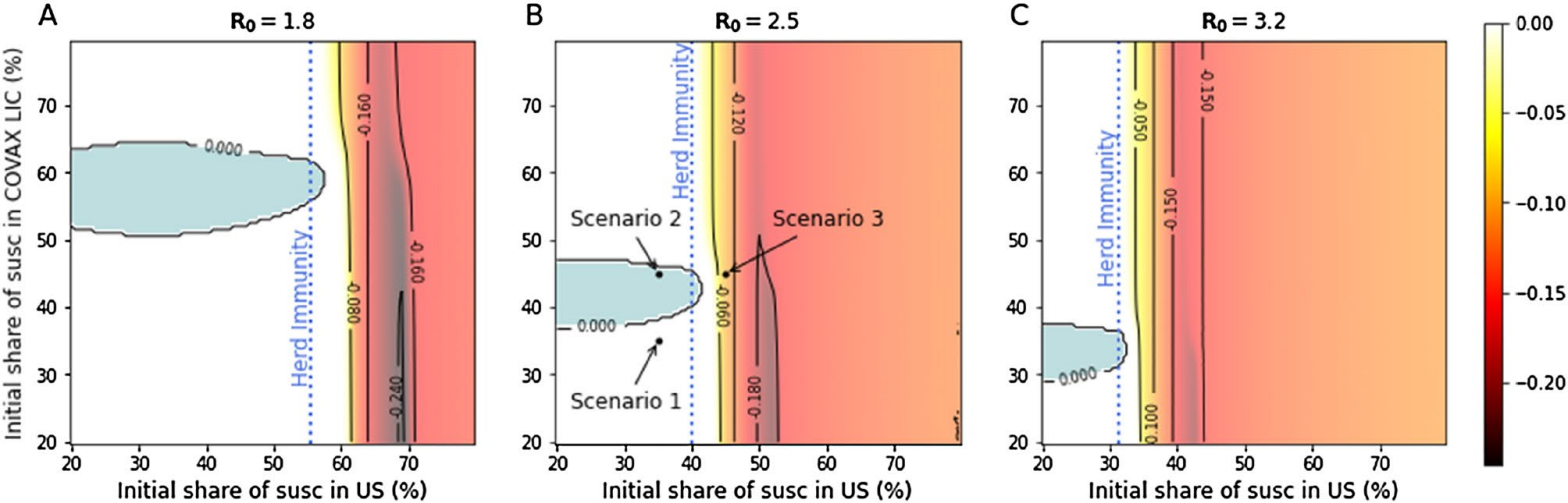
Share of individuals reached by the infection in the US, difference between uncooperative and cooperative scenarios. Difference between the share of infected in the US after 60 mln extra doses are distributed in the US (uncooperative scenario) and the share of infected in the US after the same number of doses is distributed in COVAX-LIC (cooperative scenario) for different values of ${R}_{0}$ and susceptibility levels. Positive numbers (in blue) indicate a lower share of infected in the cooperative scenario. The shares of infected in the US are estimated using a SIR compartmental model and consider the whole evolution of contagions over the time span of 10 years. As initial condition, at time 0 we set the number of infected individuals equal to 1000 in COVAX-LIC and 0 everywhere else. (**A**) Difference in shares of infected, under the assumption that *R*_0_ = 1.8. (**B**) As (**A**), but assuming *R*_0_ = 2.5. (**C**) As (**A**), but assuming *R*_0_ = 3.2. Each point corresponds to a different combination of share of susceptible at time 0 in the USA and in the COVAX-LIC. In (**B**), Scenario 1 to 3 refer to panels (**A**) to (**C**) in Fig. 2.

Figure 3 extends the analysis done in Fig. 2 to a wider set of combinations of susceptibility levels in the US and in the COVAX-LIC countries. The x-axis is the level of susceptibility in the US and the y-axis is the level of susceptibility in the COVAX-LIC countries. The plots display, for three different values of ${R}_{0}$, level curves for the difference in cases between the two distribution strategies with 60 million vaccine doses: keeping them in the US or distributing them abroad. We show that there are a number of circumstances, those shown in blue in the plots, in which the US would be better off by using those doses abroad rather than internally when considering only the benchmark of domestic US cases. As Fig. 3 shows, for a high enough share of susceptible in the US, the uncooperative strategy would guarantee a lower number of domestic infections. This is especially true in the dark shaded area, corresponding to the region where keeping the extra doses for the local population would allow the US to reach herd immunity. In blue, we highlight regions in which the cooperative strategy would lead to a smaller number of infections in the US. This is due to two factors. First, these are regions in which the US has already reached herd immunity so the marginal return to additional vaccinations is relatively low (see Methods section). Additionally, other countries are close enough to herd immunity that these additional doses allow them to achieve herd immunity and prevent sustained domestic outbreaks (Supplementary Fig. S4 extends the analysis to COVAX AMC lower and middle income countries).

## Discussion

Overall, our results show that once the US (home country) reaches herd immunity, it may be more effective to reduce domestic infections by engaging in cooperative vaccination distribution, sharing doses with other countries, rather than behaving uncooperatively and using the vaccines only domestically. This happens because the number of infections at home depends on two factors and how they interact. First, it depends on the number of infected individuals who reach the country from abroad. This quantity is a function of the share of immunizations abroad and of the network of connections, which we consider as fixed. A higher share of immunizations abroad would reduce the expected number of cases in these countries and therefore it would also reduce the expected number of infected individuals who may reach the home country. Second, the number of infections at home depends on the share of vaccinated individuals at home. A higher share of immunized people in the home country not only reduces the number of those who can get infected and can thus increase circulation, but it also reduces the share of susceptible individuals who may be reached by the disease. This is the fundamental trade-off that we highlight in this paper.

By sharing vaccine doses, a country benefits from a reduction in the number of cases from abroad at the expense of more contagion once an infected individual reaches the home country. These two effects—the number of cases from abroad and the spread of the disease at home-are not linear functions of the number of immunizations. Instead, they also depend on the initial level of immunized individuals both domestically and abroad. Additional doses have a greater impact on the country in which they are distributed if the country is below, but close to, the herd immunity threshold. If a country is already above herd immunity, however, further domestic vaccinations have a much smaller effect in preventing sustained domestic transition. This explains why we find parameter regions in which the benefits from reducing contagions abroad dominate, and the home country–in our example, the US–would benefit from giving vaccine doses to other countries (as shown in Figs. 2 and 3). Although we take US as an example, our results generalize to other countries as well.

We approach the question of optimal vaccine distribution from a country’s self-interest perspective, which to our knowledge has not been attempted before. We build on prior work by Chinazzi et al.^13^ who argued that equity justifies cooperative vaccine distribution because it would reduce by a factor of two overall global deaths. Our results show that in addition to reducing total global deaths, it may also be in a country’s self-interest, defined narrowly as its domestic disease burden, to share vaccines once it has achieved a certain threshold to prevent infections domestically. The use of a simple but analytically solvable network SIR model allows us to make general predictions and compare many different scenarios.

Our model parameters are based on current understandings of SARS-CoV-2 and vaccine efficacy, which may change as more information becomes available. However, our model is robust to different specifications of these parameters. Varying ${R}_{0}$ between 1.5 and 3.2 and the vaccine efficacy between 0.9 and 1 did not change the results. This work was undertaken before the evolution of the delta variant with a higher ${R}_{0}$ value. However, the broader principle of self-interest still holds, although a country would need to have a higher number of people vaccinated (because achieving herd immunity becomes harder) before the cooperative strategy would generate a domestic benefit, which is shown in Fig. 3 (lower left region colored blue).

We use travel data from 2019 for network specifications. This does not account for reduced travel and travel restrictions in place during the pandemic. However, as our goal is to evaluate what is in a country’s self-interest, including economic interest, a pre-pandemic specification is more relevant for understanding how to maximize a country’s well-being. Importantly, as vaccination becomes widespread in the US, a cooperative distribution strategy will allow the US to keep its international borders open and avert economic losses inherent to the border closing. If travel remains below the levels modeled here, then it would reduce somewhat the return to foreign vaccine allocation.

Several other factors, beyond those considered in our model, may also motivate allocating vaccines abroad. First, it may be that distributing vaccines abroad may reduce total worldwide caseloads, while we consider only the effects of allocations on domestic infections. Second, there may be other self-interested arguments for sharing vaccines globally. For example, sharing vaccines abroad may reduce the probability that vaccine avoidant variants emerge or reduce economic losses caused by trade disruptions associated with foreign cases^14^. Additionally, our model does not account for differences in the cost of vaccine delivery across countries or for differences in the speed of administration. Assuming foreign delivery was more costly or slower, it would slightly reduce the incentive to allocate vaccines abroad. Nevertheless, we note that the speed of vaccine delivery has slowed in many countries with relative high levels of immunization, while at the same time we see evidence of higher vaccine acceptance in low- and middle-income countries^15^. From a public policy perspective, this consideration highlights the importance of thinking not just about the availability of vaccines, but also about support for the infrastructure to transport, store, and deliver the vaccine to end users.

## Methods

### Single country SIR model

We start from the standard SIR model^16^. The equations of the SIR model are

1 $$\begin{array}{*{20}c} {\frac{dS}{{dt}} = - \beta IS} \\ \end{array}$$

2 $$\begin{array}{*{20}c} {\frac{dI}{{dt}} = \beta IS - \nu I} \\ \end{array}$$

3 $$\begin{array}{*{20}c} {\frac{dR}{{dt}} = \nu I} \\ \end{array}$$

where *S*, *I* and *R* are respectively the fraction of susceptible, infected, and recovered people. *β* and *ν* are respectively the infectiousness of and recovery rate from the disease. Note that *β* and *ν* depend on policy choices that are, for instance, mask wearing, social distancing, quarantine measures, and health care expenditures. Also note that, if there is a vaccination that guarantees temporary immunization, also the level $S_{0}$ of *S* that we start from is a policy choice: how many people to maintain vaccinated in steady state.

We call $R_{0} = \beta /\nu$. The system, in a situation in which *I* = 0 and *R* ≥ 0, is stable to perturbations if *S(t* = *0)* < 1*/R*_0_, which is to say that *R* > 1 − 1*/R*_0_. We assume R_0_ > 1, to consider the interesting case where computations are not trivial, and we call $P_{c} = 1 - 1/R_{0}$ the threshold for *herd immunity*.

Starting from a stable situation, with *S* = *S*_0_, we can compute the number of additional infection (leading eventually to recoveries) if there is an initial shock *I(t* = *0)*. Starting by dividing (2) by (1), We have $\frac{dI}{{dS}} = - 1 + \frac{1}{{SR_{0} }}$, which gives

4 $$\begin{array}{*{20}c} {dI = \left( { - 1 + \frac{1}{{SR_{0} }}} \right)dS} \\ \end{array}$$

Integrating $\mathop \smallint \limits_{0}^{\infty } dI = \mathop \smallint \limits_{0}^{\infty } \left( { - 1 + \frac{1}{{SR_{0} }}} \right)dS$, we obtain $\left[ I \right]_{0}^{\infty } = \left[ { - S + \frac{\log \left( S \right)}{{R_{0} }}} \right]_{0}^{\infty }$. This in turn becomes $I\left( {t = 0} \right) = \phi \left( {S\left( {t = 0} \right)} \right) - \phi \left( {S_{\infty } } \right)$, if we call $\phi \left( x \right) = x - \frac{\log \left( x \right)}{{R_{0} }}$.

So, how many additional people will get infected can be obtained (in general, non–analytically) from this $\phi \left( x \right)$ function. The infection can spread to a limited part of the population (and not many) only if $\phi$ is decreasing in *S*_0_ (which is just the previous condition *S*_0_ < 1*/R*_0_, which means starting from a stable situations).

The multiplicative effect of a small shock, when starting from a stable steady, can be computed as $1/\phi^{{\prime }}$, which we define as ${\updelta } = \frac{{S\left( {t = 0} \right)R_{0} }}{{1 - S\left( {t = 0} \right)R_{0} }}$. This number is greater when we are close to the minimal herd immunity (at the limit $S\left( {t = 0} \right)$ → 1*/R*_0_ it is infinite) and goes to 0 as $S\left( {t = 0} \right)$ → 0. This means that if the system is stable, but close to the minimal level of herd immunity, a very small shock can still propagate a lot. In this way, we relate the propagation of small shocks to the three policy variables that a country has: *β*, *ν* and $S\left( {t = 0} \right)$. *δ* is increasing in *β* and *R*_0_, and it is decreasing in *ν*. Note also that *δ* can be obtained directly from Eq. (4), without taking the integrals. This is because we are just approximating linearly the effects of small shocks.

### Network of countries

Consider a situation where there are *n* connected countries. Each country *j* has its own $R_{0,j} = {\upbeta }_{j} /{\upnu }_{j}$ and its own $P_{c,j}$. All these variables are heterogeneous across countries because they depend on the medical and social policies that are chosen.

Consider a network **G** where $g_{k,j}$ is the number of people moving from country *k* visiting country *j*, divided by the population of country *j*. **G** is null in the diagonal.

In the network, Eq. (2) becomes $\frac{{dI_{j} }}{dt} = {\upbeta }_{j} \left( {I_{j} + \mathop \sum \limits_{k} I_{k} g_{kj} } \right)S_{j} - {\upnu }_{j} I_{j}$. In this case we can include the network effect in Eq. (4), obtaining:

5 $$\begin{array}{*{20}c} {dI_{j} = \left( { - 1 + \frac{1}{{S_{j} R_{0,j} }} - \mathop \sum \limits_{k = 1}^{n} g_{kj} \frac{{I_{k} }}{{I_{j} }}} \right)dS_{j} } \\ \end{array}$$

Let us call **D** the diagonal matrix where the diagonal element $d_{jj}$ is $\frac{1}{{S_{j} R_{0,j} }}$. **I** is the identity matrix. Then, under the assumptions that for small initial shocks we have $\frac{{I_{k} }}{{I_{j} }} \simeq 1$, we can write Eq. (5) in vectorial form as $d\vec{I} = \left( { - I + D - G} \right)d\vec{S}$. This can be inverted, to find with a linear approximation how many new infected (eventually recovered) will result from an initial shock *I*(*t* =  0). That is $d\vec{S} = \left( { - {\varvec{I}} + {\varvec{D}} - {\varvec{G}}} \right)^{ - 1} d\vec{I}$. So, matrix (− **I** + **D** − **G**)^−1^ tells us how the level of infection is from each country to any other country. One way to interpret this expression is with a discrete time approach. The first–order effect of the contagion $d\vec{I}$ is $\left( {{\varvec{D}} - {\varvec{G}}} \right)\user2{d}\vec{I}$: the diagonal element accounts for recoveries, while ${\varvec{G}}$ has all the network effects (we have a minus sign because this is the decrease in susceptible people). But there is a second order effect in the second step, which is $\left( {{\varvec{D}} - {\varvec{G}}} \right)^{2} \user2{d}\vec{I}$; and then ${ }\left( {{\varvec{D}} - {\varvec{G}}} \right)^{3} \user2{d}\vec{I}$, and so on. This amounts to

6 $$\begin{array}{*{20}c} {\mathop \sum \limits_{t = 1}^{\infty } \left( {D - G} \right)^{t} d\vec{I} = \left( { - {\varvec{I}} + {\varvec{D}} - {\varvec{G}}} \right)^{ - 1} d\vec{I}} \\ \end{array}$$

Formula (6) is equivalent to the formula for Bonacich centrality^17,18^.

To have a clearer interpretation, let $\vec{m}$ be the inhabitants of each country and $Diag\left( {\vec{m}} \right)$ be the diagonal matrix in which the diagonal consists of vector $\vec{m}$. With this notation in mind, the matrix

7 $$\begin{array}{*{20}c} {Diag\left( {\vec{m}} \right)\left( { - {\varvec{I}} + {\varvec{D}} - {\varvec{G}}} \right)^{ - 1} Diag\left( {\vec{m}} \right)^{ - 1} } \\ \end{array}$$

tells how many people in each column–country will be infected if one person is infected in row–country.

### Measures of risk

We can start from the matrix expressed in Eq. (7) to identify some intuitive measures of risk for the countries. To begin, consider that if we set $d\vec{I}$ to a vector $\vec{1}$ of all 1’s, we obtain a vector $\vec{c} = \left( { - {\varvec{I}} + {\varvec{D}} - {\varvec{G}}} \right)^{ - 1} \vec{1}$*.* A country *j* that has *c*_*j*_ relatively higher with respect to other countries, will be more at risk of propagation of the infection among its population. Note that, without network effects, this expression will give us $c_{j} = {\updelta }_{j} = \frac{{S_{0,j} {\uprho }_{j} }}{{1 - S_{0,j} {\uprho }_{j} }}$, which was the result in isolation. So, this vector tells us which country is more susceptible to the propagation of the infection.

This approach allows us to study how a country can reduce its own *c*_*j*_ playing at one side with the medical parameters *β*_*j*_ (e.g., lockdown measures and policies on masks), *ν*_*j*_ (e.g., better cures, isolation of infected people) and *R*(*t* = *0*)_*,j*_ (number of vaccinated people, once a vaccine is available)—all these factors affect matrix **D**; at the other side with **G** (possibly closing borders, or controlling people entering the country). Vector $\vec{c}$ includes all these aspects and takes into account the externalities between countries. The problem with vector $\vec{c}$ is that it has not an immediate intuitive interpretation. Now, to express more intuitive measures, we need a vector $\vec{p}$ assigning the probability that a first positive infect appears in each country. We do this by obtaining two vectors.

First, we consider which country is more at risk. $Diag\left( {\vec{m}} \right)\left( { - {\varvec{I}} + {\varvec{D}} - {\varvec{G}}} \right)^{ - 1} Diag\left( {\vec{m}} \right)^{ - 1} \vec{p}$ is a column vector that says how at risk each country is. Entry *i* of this vector says, in expectation, how many people will become infected in country *i* if a first person is infected in any of the countries. A simple candidate for $\vec{p}$ is $\vec{m}/\left( {\vec{m} \cdot \vec{1}} \right),$ where $\vec{1}$ is a vector of all 1’s and · is the vector multiplication. This assumes that the probability that an infection starts is a country is simply proportional to its population. Under this assumption, the previous vector is

8 $$\begin{array}{*{20}c} {Diag\left( {\vec{p}} \right)\left( { - {\varvec{I}} + {\varvec{D}} - {\varvec{G}}} \right)^{ - 1} } \\ \end{array}$$

We call this the risk measure.

Second, we consider which country is more dangerous.

9 $$\begin{array}{*{20}c} {\vec{1}^{{\prime }} Diag\left( {\vec{m}} \right)\left( { - {\varvec{I}} + {\varvec{D}} - {\varvec{G}}} \right)^{ - 1} Diag\left( {\vec{m}} \right)^{ - 1} } \\ \end{array}$$

is a row vector that says how dangerous each country is. Entry i in this vector tells us how many infections will be caused by a person getting infected in country i. We call this the danger measure. Note that, even if matrix G is symmetric, risk measure from (8) and danger measure from (9) are different, because of the different population size of the countries. One could also change the formula in (9), so that the diagonal elements are removed from (− I + D − G)^−1^: in this case we would have a danger to others measure, because we should not compute those that eventually become infected in the country where the disease originated.

These results are valid for small effects, that are obtained when all countries have herd immunity. To study the effects when a country is below herd immunity we must rely on simulations.

### The marginal effect of vaccinations

We proceed by simulating the system described by Eqs. (1), (2), and (3). The data on population were retrieved from The World Bank. We first simulate the model for the US, to study which fraction of the population would be eventually reached in case of an initial shock of 1000 people infected at time 0. We let the model run for 10 years and ignore the rest of the network. The fraction of population eventually reached by the infection is a function of the initial share of susceptible.

This analysis allows to study the reduction in the number of infections that can be obtained by reducing by 30 million the number of susceptible individuals in the country, this can be interpreted as the marginal effect of 60 million doses of vaccine. This number is function of the initial share of susceptible individuals. When the initial share of susceptible individuals is low, the reduction in the number of infections is close to 0, as the initial shock would propagate little across the population. The number grows and reaches a peak that corresponds to the herd immunity threshold. In this point the extra doses of vaccine would have the largest impact as they would allow the system to reach herd immunity (Supplementary Fig. S3).

### Simulations with data on flights

We proceed by simulating the system described by Eqs. (1), (5), and (3) using data on flights for the year 2019. Data were obtained from ICAO and contain information on the number of passengers in international flights. In the network we therefore use countries as nodes. Links represent the average daily number of passengers going from one country to another. A graphical representation of this network is in Supplementary Fig. S1. Supplementary Fig. S2 shows the same network, where nodes sizes are proportional to the risk measure defined above.

We set *γ* = 1*/*18 that corresponds to an average duration of illness of 18 days^19^. We simulate the model under various assumptions for the level of susceptible individuals in each country. When considering countries belonging to COVAX-LIC, we aggregate so that they appear as a single unit. To do that, we take the total population summing across all countries. For the network, we perform a similar operation, considering all flights that connect any of the COVAX-LIC countries with any other country in the network. To solve the system of differential equations we use SciPy^20^ integrate.solve ivp solver, using Adams/BDF method. Matplotlib^21^ was used to make Figs. 1, 2, 3, S3, and S4. Gephi^22^ was used to make Figures S1 and S2.

## Supplementary Information

Supplementary Information.

## Data Availability

All code will be made publicly available to download. Data on flight connections are restricted and can be downloaded from the provider’s website.

## References

[1] Dong E, Du H, Gardner L (2020). An interactive web-based dashboard to track COVID-19 in real time. Lancet Inf. Dis..

[2] Moreland A, Herlihy C, Tynan MA (2020). Timing of state and territorial COVID-19 stay-at-home orders and changes in population movement-United States, March 1–May 31, 2020. MMWR Morb. Mortal. Wkly Rep..

[3] Chudik A, Mohaddes K, Pesaran MH, Raissi M, Rebucci A (2020). A counterfactual economic analysis of Covid-19 using a threshold augmented multi-country model. NBER Work. Pap..

[4] Cutler DM, Summers LH (2020). The COVID-19 pandemic and the $16 trillion virus. JAMA.

[5] Nicola M (2020). The socio-economic implications of the coronavirus pandemic (COVID-19): a review. Int. J. Surg..

[6] Goolsbee A, Syverson C (2020). Fear, lockdown, and diversion: comparing drivers of pandemic economic decline. J. Public Econ..

[7] Atkeson A (2020). What will be the economic impact of COVID-19 in the US? Rough estimates of disease scenarios. NBER Working Pap..

[8] Duke Global Health Innovation Center. (2021); Launch and Scale Speedometer. Duke University. Retrieved from: https://launchandscalefaster.org/covid-19/vaccineprocurement. Accessed 26 Apr 2021.

[9] Gavi The Vaccine Alliance, COVAX Explained, (2020). Retrieved from: https://www.gavi.org/vaccineswork/covax-explained. Accessed 26 Apr 2021.

[10] Fidler DP (2020). Vaccine nationalism's politics. Science.

[11] Chinazzi M (2020). The effect of travel restrictions on the spread of the 2019 novel coronavirus (COVID-19) outbreak. Science.

[12] Fine P, Eames K, Heymann DL (2011). “Herd immunity”: a rough guide. Clin. Infect. Dis..

[13] Chinazzi M (2020). Estimating the Effect of Cooperative Versus Uncooperative Strategies of COVID-19 Vaccine Allocation: A Modeling Study.

[14] Çakmaklı, C., Demiralp, S., Kalemli-Özcan, Ṣ., Yeşiltaş, S., & Yıldırım, M. A. () The economic case for global vaccinations: an epidemiological model with international production networks. *NBER* Working Paper No. 28395 (2021).

[15] Solís Arce JS, Warren SS, Meriggi NF (2021). COVID-19 vaccine acceptance and hesitancy in low- and middle-income countries. Nat. Med..

[16] Heesterbeek JAP (2002). A brief history of *R*_0_ and a recipe for its calculation. Acta Biotheor..

[17] Bonacich P (1987). Power and centrality: a family of measures. Am. J. Sociol..

[18] Dequiedt V, Zenou Y (2017). Local and consistent centrality measures in parameterized networks. Math. Soc. Sci..

[19] Wang H (2020). Cai Phase-adjusted estimation of the number of Coronavirus Disease 2019 cases in Wuhan, China. Cell Discov..

[20] Virtanen P, Gommers R, Oliphant TE (2020). SciPy 1.0: fundamental algorithms for scientific computing in Python. Nat. Methods.

[21] Hunter JD (2007). Matplotlib: a 2D graphics environment. Comput. Sci. Eng..

[22] Bastian M., Heymann S. & Jacomy M. Gephi: an open source software for exploring and manipulating networks. *International AAAI Conference on Weblogs and Social Media* (2009).

