# Supplementary material for: National interest may require distributing COVID-19 vaccines to other countries
Source: Sci Rep. 2021 Sep 14;11:18253. doi: 10.1038/s41598-021-97544-5 (PMC8440602; doi:10.1038/s41598-021-97544-5)
Supplement: Supplementary file 1 — Supplementary Information. [file 41598_2021_97544_MOESM1_ESM.docx]

Supplementary Material for:

 National interest may require distributing COVID-19 vaccines to other countries

**Authors:** Tiziano Rotesi*, Paolo Pin, Maria Cucciniello, Amyn A. Malik, Elliott E. Paintsil, Scott E. Bokemper, Kathryn Willebrand, Gregory A. Huber, Alessia Melegaro, Saad B. Omer

**COVAX AMC countries**

**COVAX AMC Lower Income Countries**: Afghanistan, Benin, Burkina Faso, Burundi, Central African Republic, Chad, Dem. Rep. of the Congo, Eritrea, Ethiopia, Gambia, Guinea, Guinea-Bissau, Haiti, Liberia, Madagascar, Malawi, Mali, Mozambique, Nepal, Niger, Rwanda, Sierra Leone, Somalia, South Sudan, Syrian Arab Republic, Tajikistan, Togo, Uganda, United Republic of Tanzania, Yemen Rep.

**COVAX AMC Lower and Middle Income Countries**: Afghanistan, Algeria, Angola, Bangladesh, Benin, Bhutan, Bolivia, Burkina Faso, Burundi, Cabo Verde, Cambodia, Cameroon, Central African Republic, Chad, Comoros, Congo, Cote d’Ivoire, Dem. Rep. of the Congo, Djibouti, Egypt, El Salvador, Eritrea, Eswatini, Ethiopia, Gambia, Ghana, Guinea, Guinea-Bissau, Haiti, Honduras, India, Indonesia, Kenya, Kiribati, Kyrgyzstan, Lao PDR, Lesotho, Liberia, Madagascar, Malawi, Mali, Mauritania, Micronesia Fed. Sts., Mongolia, Morocco, Mozambique, Myanmar, Nepal, Nicaragua, Niger, Nigeria, Pakistan, Papua New Guinea, Philippines, Rwanda, Sao Tome and Principe, Senegal, Sierra Leone, Solomon Islands, Somalia, South Sudan, Sri Lanka, Sudan, Syrian Arab Republic, Tajikistan, Timor-Leste, Togo, Tunisia, Uganda, Ukraine, United Republic of Tanzania, Uzbekistan, Vanuatu, Viet nam, West Bank and Gaza, Yemen, Rep., Zambia, Zimbabwe.

**Supplementary Figures**


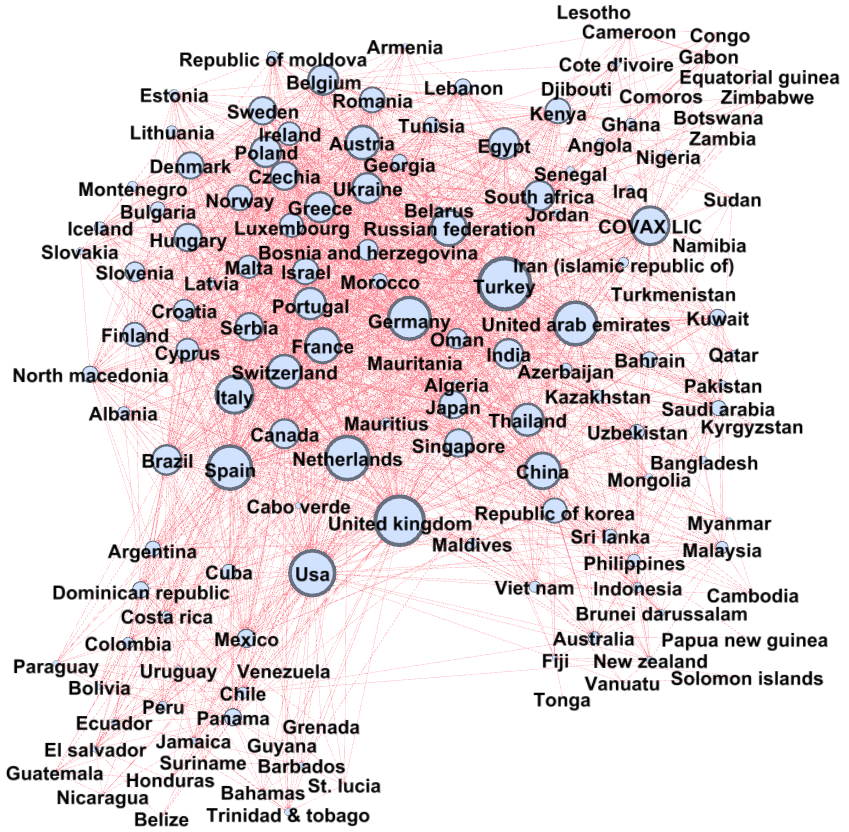


**Fig S1. Network of Flights**. Nodes are countries and the size depends on the total inflow of passengers reaching each country. A direct link denotes the existence of a direct commercial flight between the two countries.


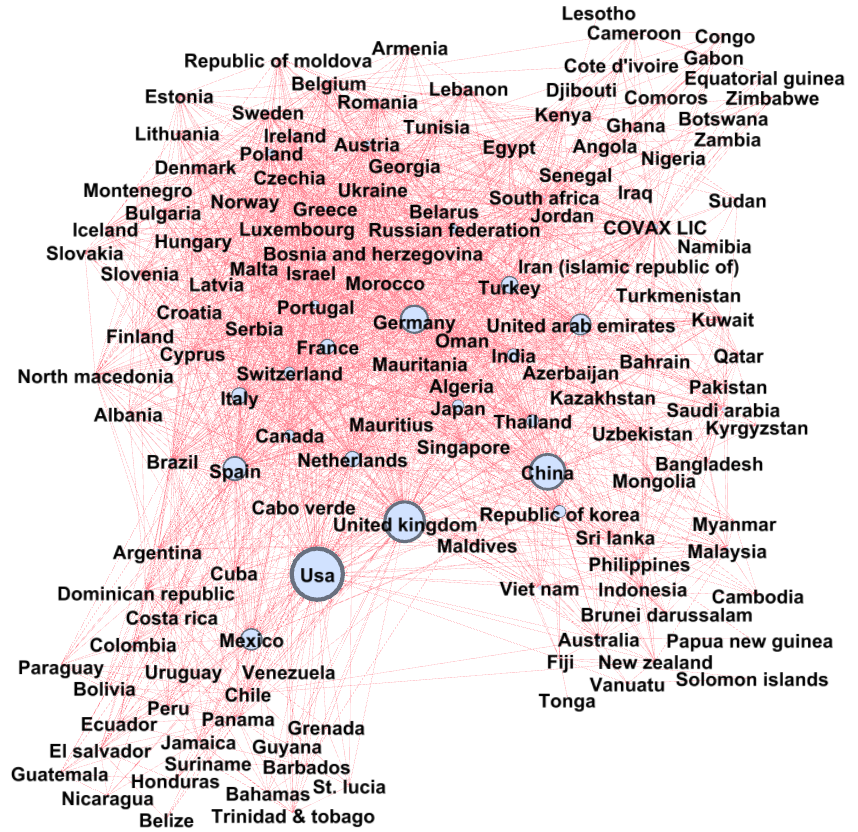


**Fig S2. Network of Flights**. Nodes are countries and the size are proportional to the risk measure, calculated assigning a share of susceptible equal to 35% to every country. A direct link denotes the existence of a direct commercial flight between the two countries.


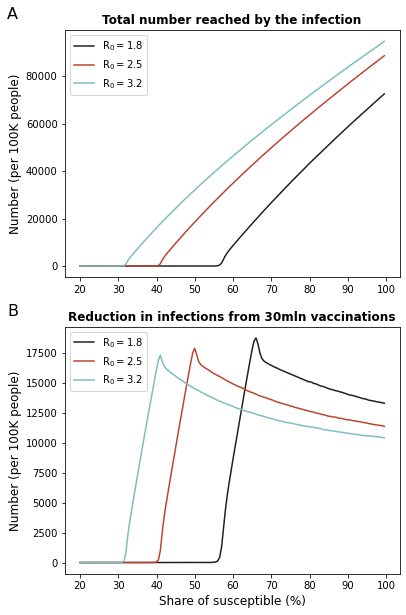


**Fig S3. Marginal effects of additional vaccinations. (A)** The figure shows the number of people eventually reached by the infection after 1000 individuals get infected at time 0. The number is expressed as a function of the initial share of susceptible individuals. **(B)** The figure shows the reduction in the number of infections that would be determined by 60 million additional doses (30 million immune individuals) done before the start of the contagion at time 0. This number varies as it depends on the spread of the disease determined by the share of susceptible individuals.


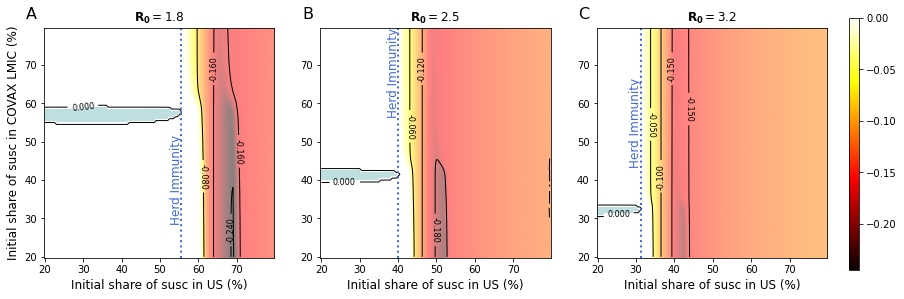


**Fig S4.** Share of individuals reached by the infection in the US, difference between uncooperative and cooperative scenarios. Difference between the share of infected in the US after 60mln extra doses are distributed in the US (uncooperative scenario) and the share of infected in the US after the same number of doses is distributed in COVAX AMC lower and middle income countries (cooperative scenario) for different values of *R*_0_ and susceptibility levels. Positive numbers (in blue) indicate a lower share of infected in the cooperative scenario. The shares of infected in the US are estimated using a SIR compartmental model and consider the whole evolution of contagions over the time span of 10 years. As initial condition, at time 0 we set the number of infected individuals equal to 1000 in COVAX AMC lower and middle income countries and 0 everywhere else. (A) Difference in shares of infected, under the assumption that *R*_0_ = 1*.*8. (B) As (A), but assuming *R*_0_ = 2*.*5. Each point corresponds to a different combination of share of susceptible at time 0 in the US and in the COVAX AMC lower and middle income countries. (C) As (A), but assuming *R*_0_ = 3*.*2.
